# Consumption of a Branched-Chain Amino Acids-Containing Sports Beverage During 21 km of Running Reduces Dehydration, Lowers Muscle Damage, and Prevents a Decline in Lower Limb Strength

**DOI:** 10.3390/nu16223799

**Published:** 2024-11-05

**Authors:** Zhuoying Liang, Yiheng Liang, Chengnan Zhang, Xueyuan Zhao, Junqiang Qiu

**Affiliations:** 1 Department of Exercise Biochemistry, Exercise Science School, Beijing Sport University, Beijing 100084, China; liangzhuoying@bsu.edu.cn (Z.L.); yihengliang@bsu.edu.cn (Y.L.); 2023992990@bsu.edu.cn (C.Z.); zhaoxueyuan@bsu.edu.cn (X.Z.); 2 Beijing Sports Nutrition Engineering Research Center, Beijing 100084, China

**Keywords:** hydration status, electrolyte balance, branched-chain amino acids, endurance exercise performance

## Abstract

Objectives: The purpose of this study was to examine the acute effects of branched-chain amino acids (BCAAs)-containing electrolyte beverage (AE) on water–electrolyte balance, muscle damage, time to finish the final 5 km, and muscle strength compared to a standard commercially available carbohydrate–electrolyte sports beverage (CE), pure water (W), and no rehydration (N). Methods: Fourteen trained male participants (20 ± 2 years old) completed four randomized 21 km running trials. The participants were instructed to consume their drink (150 mL W, 150 mL CE, or 150 mL AE) or no rehydration (N) at 5 km, 10 km, and 15 km. Body mass and muscle strength were assessed, and blood samples were collected before and after exercise. Perceptual scales were administered during and after running. Blood electrolyte levels (sodium, potassium, and chloride) and creatine kinase (CK) concentration were analyzed. Results: The change in plasma volume with AE was significantly smaller than that with N (*p* < 0.05). Consuming AE maintained the best potassium balance (*p* < 0.05). Twenty-four hours after exercise, serum CK concentrations significantly elevated in N, W, and CE (*p* < 0.05), but did not reach statistical significance in the AE group (*p* > 0.05). Compared to N, consuming AE resulted in significantly less soreness 24 h after exercise (*p* < 0.05). There was no difference in time to finish the final 5 km (*p* > 0.05). Maximal voluntary isometric force output was significantly lower after exercise with N and W (*p* < 0.05) but not with CE or AE (*p* > 0.05). Conclusions: Consuming a BCAAs-containing sports beverage during a 21 km run can help reduce dehydration, maintain potassium balance, lower muscle damage, and prevent the decline in lower limb strength after 21 km running.

## 1. Background

Long-distance running events, such as full and half marathons, are popular worldwide, attracting athletes of all levels. During extended periods of high-intensity exercise, the body loses large amounts of fluids and electrolytes through sweat, resulting in a significant decrease in body weight, which is a clear indication of dehydration [1]. Dehydration not only impairs athletic performance but also poses several health risks, including electrolyte imbalances, muscle cramps, and increased fatigue [2,3]. Sports drinks, typically carbohydrate–electrolyte beverages, have long been a key strategy for replenishing fluids and electrolytes lost during exercise [4]. Electrolytes, such as sodium, potassium, and chloride, play an essential role in regulating fluid balance within extracellular and intracellular fluid compartments and are crucial for preventing exercise-associated muscle cramps and maintaining optimal cell function [5]. Therefore, appropriate supplementation during exercise is crucial. Proper supplementation helps maintain water–electrolyte balance and reduce muscle damage.

In recent years, some sports electrolyte beverages have incorporated amino acids into their formulations [6], and it is reported that amino acids can enhance fluid replacement [7,8]. Furthermore, the repetitive strain from running can lead to varying degrees of exercise-induced muscle damage, which may exacerbate fatigue and hinder recovery [9,10,11]. Branched-chain amino acids (BCAAs), which include leucine, isoleucine, and valine, are well known for their roles in muscle protein synthesis and reducing muscle damage and post-exercise soreness [12,13]. The incorporation of BCAAs into sports beverages has emerged as a promising innovation. However, there is not unanimous agreement among studies regarding their effectiveness in enhancing performance or reducing muscle damage [14,15], particularly when BCAAs were incorporated as an ingredient in sports drinks, as their impact is not well defined. Additionally, BCAAs, especially leucine, have a naturally bitter taste, which can negatively impact the flavor profile of sports drinks [16]. Owing to constraints in flavor profile and solubility, sports beverages can only incorporate minimal quantities of BCAAs. For BCAAs to exert noticeable effects, a certain dosage must be achieved, but the minimum effective dose of BCAAs is not clear [13].

Given that the practical application effects of BCAAs as an ingredient in sports drinks remain controversial, this study aims to investigate the effects of a novel sports beverage with BCAAs on hydration status, electrolyte balance, muscle damage, and performance, compared to a traditional carbohydrate–electrolyte drink and pure water. We hypothesized that the consumption of this beverage during running can help maintain water–electrolyte balance and reduce muscle damage.

## 2. Methods

### 2.1. Participants and Ethical Approval

This study was conducted with prior review and approval from the Sports Science Experiment Ethics Committee of Beijing Sports University (No. 2024079H).

Young male adults with endurance training experience were recruited from the university, meeting the following criteria: (1) aged 18–25 years old; (2) a normal BMI range (18.5–24.9); (3) engaged in endurance training for more than 8 h per week; (4) healthy individuals without clinically diagnosed diseases; (5) no smoking, alcohol abuse, or other unhealthy habits; (6) had not participated in any clinical or nutritional research trials within the last month; (7) not participating in other sports or nutritional intervention experiments during the study; and (8) willing to follow the experimental procedures voluntarily. A total of 15 subjects who met the requirements were enrolled.

Participant characteristics are presented in Table 1. Fourteen male participants completed all trials. During the experiment, one subject did not complete all trials due to an ankle injury, and, finally, 14 subjects completed all the experimental procedures.

### 2.2. Experimental Design

**Figure 1:**
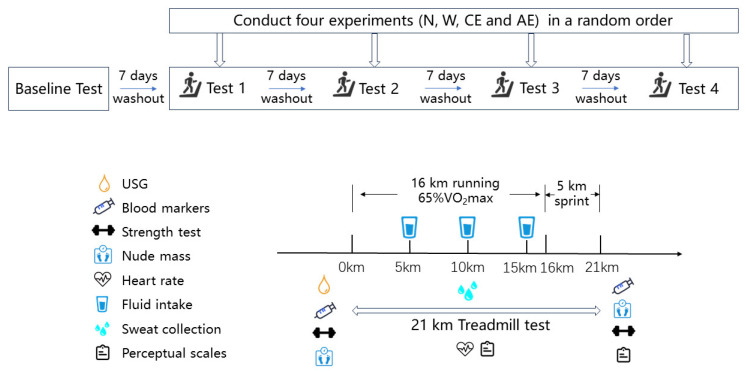
Study design. Abbreviations: USG, urine specific gravity; N, no rehydration during running; W, consuming pure water during running; CE, consuming a standard carbohydrate–electrolyte sports beverage during running; AE, consuming a branched-chain amino acids (BCAAs)-containing electrolyte beverage during running.

This is a clinical trial that is a randomized, controlled, crossover design. The timeline and testing procedures for each visit are illustrated in Figure 1 and are explained in more detail below. Initially, the volunteers underwent baseline assessments. These assessments included measurements of height, weight, and body composition, as well as a cardiopulmonary exercise test (CPET) to determine the maximum oxygen consumption (VO_2_max). This test established the speed at which they would perform the 21 km treadmill tests.

All participants were instructed to avoid consuming alcoholic or caffeinated drinks and to refrain from engaging in intense physical activities for 24 h prior to the trials. They were also asked to keep a food diary for 24 h before the first trial and to replicate their meals before each of the subsequent trials.

The experimental protocols used an electrolyte beverage fortified with BCAAs (AE, Alienergy Electrolyte Drink Professional Edition; Chi Forest, Beijing, China), a standard commercially available carbohydrate–electrolyte sports beverage (CE, GATORADE^®^ Thirst Quencher; PepsiCo, Purchase, NY, USA) and pure water (W). Additionally, please see Table 2 for a full breakdown of the ingredients included in AE and CE.

We implemented substantial measures to ensure double blinding throughout the trial. These measures included the following: (a) a designated researcher responsible for labeling and dispensing the drinks using opaque squeeze bottles; (b) participants were not informed about the composition of the liquids they consumed and could not differentiate between AE and CE based on taste; (c) each dispensed drink was assigned a random number, and during statistical analysis, the researchers did not know which number corresponded to which hydration strategy.

The 21 km treadmill test took place in the morning (08:00–10:00). Participants’ visits were scheduled to be at the same time of day (±1 h) for each visit. Each subject’s urine sample (first urine of the day) was collected to check their urine specific gravity (USG) using a portable refractometer (PAL-10S; ATAGO, Tokyo, Japan). Participants with USG values ≤ 1.025 were considered at a normal hydration state [17]. The participants had a standardized breakfast and drank 500 mL of water approximately 1.5 h before the experimental trials. Fifteen to thirty minutes before the race, they completed a standardized warm-up routine and ensured that they had urinated. Then, pre-exercise testing was completed, which included blood collection, nude body weight measurement, and lower limb strength assessment. Next, the 21 km treadmill test was conducted. Participants were required to run at a speed equivalent to 65% of their VO_2_max intensity for the first 16 km of the test.They were asked to complete the last 5 km as fast as possible and were allowed to change the treadmill speed at any time. The treadmill (Mercury 4.0, h/p/cosmos, Cologne, Germany) slope was maintained at 1% [18]. The tests were carried out in a controlled room in the morning, with a temperature of 23 ± 1 °C and a relative humidity of 35 ± 5%. Participants were asked to maintain consistent clothing for each running session.

Rehydration was scheduled at 5 km, 10 km, and 15 km to simulate an actual half-marathon race. The participants were instructed to consume their drink (150 mL W, 150 mL CE, or 150 mL AE) or no rehydration (N) at 5 km, 10 km, and 15 km. Participants’ ratings of perceived exertion (RPE), thirst, and gut comfort were recorded every 15 mins. The sweat patch (Tegaderm + Pad, 3M, St. Paul, MN, USA) was placed on the forearm to collect sweat samples during the race. Real-time heart rates were also tracked using chest strap heart rate monitors (Polar; Kempele, Oulu, Finland). Immediately following the completion of the 21 km running, participants underwent post-exercise testing, including blood markers, nude body weight, perceptual scales, and lower limb strength assessment. The timeline and each test can be visualized in Figure 1. The blood sample was taken and muscle soreness was assessed 24 h after exercise. The four treadmill tests were conducted in a random order, with participants either consuming a different randomized drink (W, CE, or AE) or not rehydrating. There was a one-week washout period between each experiment.

### 2.3. Specific Testing Procedures

#### 2.3.1. VO_2_max Assessment

VO_2_max was assessed following the methodology outlined in previous studies [19], and the corresponding speeds for different VO_2_max intensities were calculated. The VO_2_max tests were conducted on a running treadmill (h/p/cosmos Mercury 4.0, Germany) using a gas exchange analysis system (MetaMax-3B; CORTEX Biophysik GmbH, Leipzig, Germany).

The subjects began with a 5 min warm-up at a speed of 8.0 km/h. After completing the warm-up, the subjects started at a speed of 9.6 km/h for the first minute. The speed was then increased by 1.6 km/h every minute, up to a speed of 17.6 km/h. Once the speed exceeded 17.6 km/h, it was increased by 0.8 km/h every minute until the subjects were considered to have reached their maximum oxygen uptake. The treadmill incline was consistently maintained at 1%.

#### 2.3.2. Nude Body Mass Assessment

Participants were weighed nude using an electronic scale (accurate to 0.001 g) before and after exercise. They were instructed to remove their clothing, dry off any sweat, and empty their bladder. Whole-body fluid loss was calculated from the reduction in body mass that occurred during the trial. Sweat loss was estimated by accounting for the reduction in body mass and fluid intake. Any reductions in mass due to respiratory water loss or carbon loss as carbon dioxide was assumed to be negligible and consistent across trials [20].

#### 2.3.3. Measurement of Blood Markers

Hemoglobin and hematocrit were assessed by a 3-part Differential Hematology Analyzer (KX-21N; Sysmex, Kobe, Japan), and these values were used to calculate the percent change in plasma volume (%ΔPV) [21]. Two blood samples were centrifuged for 15 min at 3500 RPM at 4 °C to obtain plasma samples and serum samples. Plasma osmolality (P_OSM_) was analyzed through freezing point determination (OSMOMAT 3000; Gonotec, Berlin, Germany). Serum sodium (Na^+^), potassium (K^+^), and chlorine (Cl^−^) were assessed by an Electrolyte Analyzer (Easy Lyte Plus; MEDICA, Minnetonka, MN, USA). Creatine kinase (CK) was measured with a fully automatic immunoanalyzer (Beckman DXC 800, Beckman Coulter, Fullerton, CA, USA).

#### 2.3.4. Sweat Collection and Samples Analysis

The skin at the respective sites was shaved prior to the run. Upon completing the 10 km run, the skin of the participant’s forearm was cleaned with alcohol and pure water and the sweat patch (Tegaderm + Pad, 3M, St. Paul, MN, USA) was placed on the left dorsal forearm to collect sweat samples during the race. Patches were removed from the skin upon moderate sweat absorption (~0.5 g), but prior to saturation, as determined by visual inspection [22]. Upon removal, the absorbent pad was immediately separated from the Tegaderm™ using clean forceps and placed in an air-tight plastic tube. The sweat was separated from the patches by centrifugation. Sweat sodium (Na^+^), potassium (K^+^), and chlorine (Cl^−^) were assessed by an Electrolyte Analyzer (Easy Lyte Plus; MEDICA, Minnetonka, MN, USA). The regression equations developed by Baker et al. [22] were used to subtract the background mmol/L of electrolytes in the absorbent patches. Whole-body sweat Na^+^, K^+^, and Cl^−^ were predicted from the dorsal forearm through the prediction models developed by Baker et al. [22,23].

Electrolyte balance was calculated by subtracting the amount of electrolyte lost through sweat from the amount ingested. Negative values in fluid and electrolyte balance indicate deficits. These calculations assumed that sweat rate and sweat electrolyte concentration remained constant throughout the race, although slight variations likely occurred [24].

#### 2.3.5. 5 km Time Trials

After completing 16 km, participants were allowed to adjust the treadmill speed to complete the final 5 km as quickly as possible. The time taken to complete this segment was recorded.

#### 2.3.6. Lower Limb Strength Assessment

Participants performed three countermovement vertical jumps for maximal height on a vertical jump measurement device (GMCS-IV; Beijing Sport University Sports Engineering Research Center, Beijing, China) to assess pre-race and post-race jump height with their hands placed on their hips. The highest jump was used for statistical analysis. Participants familiarized themselves with the jump test beforehand.

Maximal voluntary isometric contraction (MVIC) of the quadriceps was assessed using a versatile knee extension device (David F-200; David GmbH, Neu-Ulm, Germany), equipped with a digital analysis module (MC-M; DAVID GmbH, Neu-Ulm, Germany) that recorded real-time and peak torque during isometric muscle contractions. Participants adjusted the seat height to ensure proper positioning, which aided in optimal muscle exertion. A strap across the waist secured the subjects to maintain a consistent trunk-thigh angle of 120° and a knee joint angle of 120°. The lever arm’s rotation axis aligned with the lateral femoral epicondyle of the participant’s right leg, and the participants adjusted the position of the lever arms. The seat height and lever arm positions were recorded to ensure consistency across all subsequent tests. Participants performed three 3 s maximal voluntary isometric knee extension trials, with a 2 min rest between each trial [25]. The highest torque recorded during these tests was used for statistical analysis.

#### 2.3.7. Perceptual Scales Assessment

The Borg 6–20 RPE scale was used to assess the participants’ perceived exertion, where a score of 6 indicates no exertion at all and 20 indicates maximal exertion. Participants’ perceived levels of thirst and gastrointestinal comfort were assessed using a Likert scale ranging from 0 to 10 (thirst: “0 = not thirsty at all” to “10 = extremely thirsty”; gastrointestinal comfort: “0 = no discomfort” to “10 = extreme discomfort”). These scale assessments were conducted every 15 min during exercise and immediately after the completion of 21 km of running. Participants evaluated their perceived level of muscle soreness using a visual analog scale (VAS). Soreness was assessed along a 10 cm scale (0 cm = no soreness, 10 cm = extreme soreness).

#### 2.3.8. Supplement Protocol

During the four running tests, the participants randomly consumed the drinks (W, CE, or AE) or no rehydration (N). Rehydration was scheduled at the distances of 5 km, 10 km, and 15 km to simulate an actual half-marathon race. At each rehydration point, participants consumed 150 mL of their drink. The drinks were labeled in equal portions and stored in squeeze bottles to prevent any loss of liquid during ingestion.

#### 2.3.9. Dietary Assessment and Control

Participants maintained daily records of their food and beverage intake using a mobile nutrient analysis app (Boohee, Information Technology Co., Ltd., Shanghai, China) the day before and on the day of the experiment. The effectiveness of the Boohee app as a dietary monitoring tool has been validated in previous research [25]. Participants consumed the same food on the day before each of the four experimental sessions, with the same requirement applied on the day of each experiment (Table 3). Dietary control on the day before the experiment aimed to avoid inconsistent sodium intake, which could affect blood sodium concentration and sweat composition. On the day of the experiment, dietary control prevented variations in protein and energy intake from impacting muscle recovery within 24 h post-exercise.

### 2.4. Statistical Analysis

We planned to detect a moderate effect size (0.3) on the primary outcome with a statistical power (1 − β) of 0.9 and an alpha error (α) of 0.05 in a repeated-measure design, resulting in a minimum requirement of eleven subjects (calculated by G*Power 3.1.9.7; software developed at Dusseldorf University, Germany). Analyses were performed using IBM SPSS Statistics version 22.0 software. Data are presented as the mean and standard deviations (SD). To assess normality, the Shapiro–Wilk test was applied initially. Repeated measures ANOVA with the Tukey post hoc test was used to statistically analyze the difference between groups. A significance level of 0.05 was set for all statistical tests. All graphs were plotted with GraphPad Prism 10 (GraphPad Software, San Diego, CA, USA).

## 3. Results

### 3.1. Hydration Status

**Figure 2:**
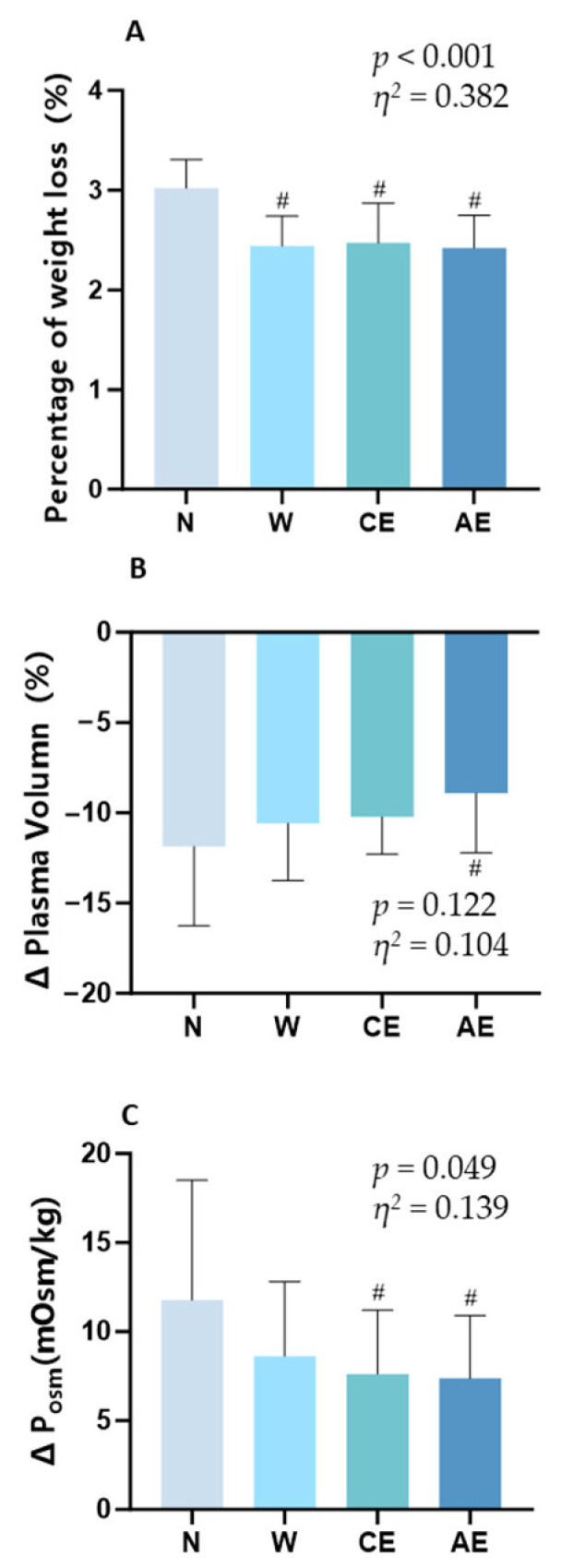
Hydration status of the participants: (**A**) percentage of nude body mass lost after 21 km running; (**B**) plasma volume changes; (**C**) plasma osmolality changes. Significance symbols: ^#^, significantly different from N, *p* < 0.05.

Figure 2 shows the hydration status of participants after exercise. The percentage of nude body mass loss in W, CE, and AE was lower than that in N (N: 3.02 ± 0.29%; W: 2.44 ± 0.30%; CE: 2.47 ± 0.40%; AE: 2.42 ± 0.33%; *p* < 0.001; *η*^2^ = 0.382, 95% CI: [0.369, 0.392]; Figure 2A). For the change in plasma volume, there were no significant differences among the groups (*p* = 0.122; *η*^2^ = 0.104, 95% CI: [0.056, 0.152]; Figure 2B), but a significant difference was detected specifically between AE and N (*p* = 0.019, Cohen’s d = 0.76, 95% CI [−0.005, 1.525]). The change in plasma volume in AE was significantly smaller than that in N (−8.91 ± 3.30% vs. −11.86 ± 4.39%). The changes in plasma osmolality before and after exercise in CE and AE were significantly lower than that in N (N: 11.77 ± 6.73 mOsm/kg; W: 8.62 ± 4.19 mOsm/kg; CE: 7.62 ± 3.59 mOsm/kg; AE: 7.38 ± 3.52 mOsm/kg; *p* = 0.049; *η*^2^ = 0.139, 95% CI: [0.002, 0.101]; Figure 2C), and there was no significant difference between N and W (*p* = 0.560).

### 3.2. Blood Measures

**Figure 3:**
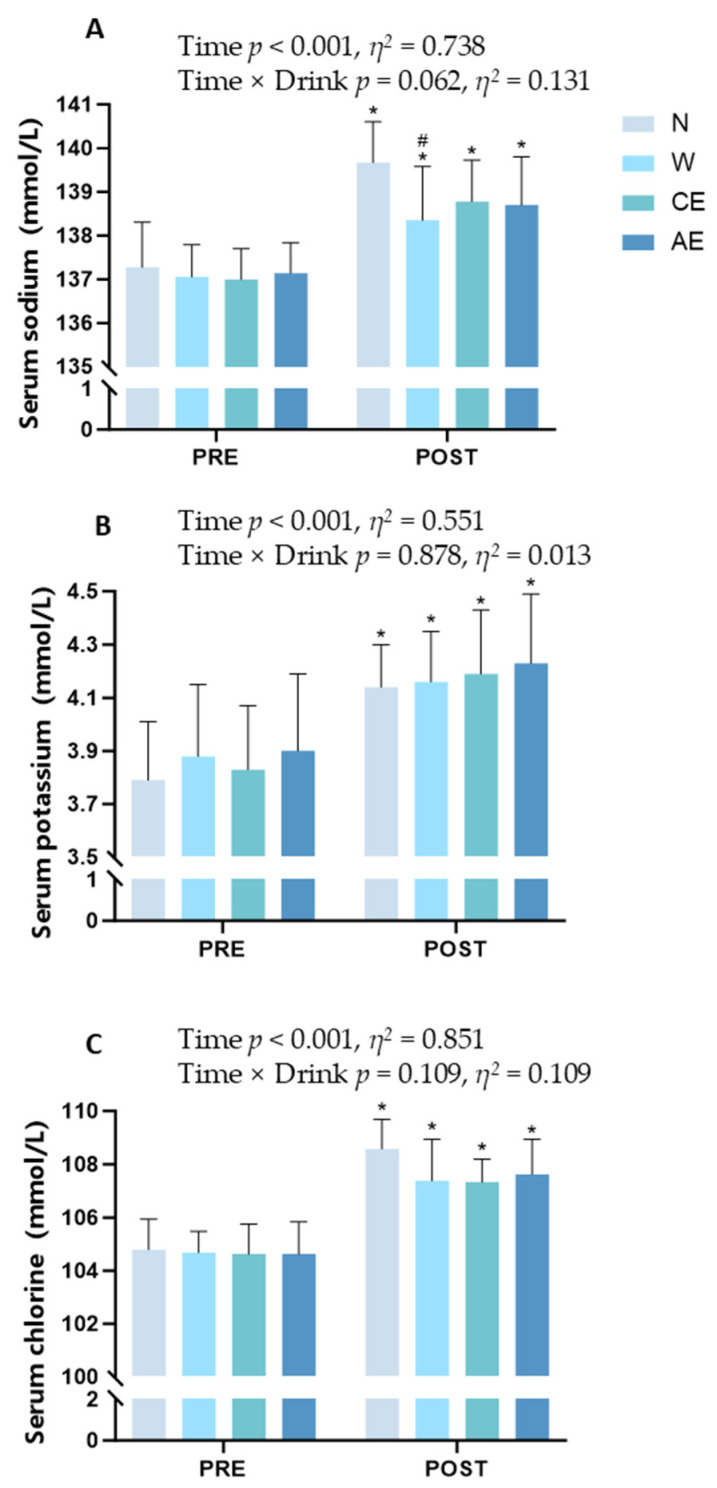
Analysis of serum electrolyte concentration before and after the exercise protocol for the four conditions evaluated: (**A**) serum sodium concentration; (**B**) serum potassium concentration; (**C**) serum chlorine concentration. Significance symbols: ^#^, significantly different from N, *p* < 0.05; *, significantly different from pre-exercise, *p* < 0.05.

The serum analysis indicated that the blood electrolyte concentration displayed a significant increase from pre-exercise to post-exercise (Figure 3). For serum sodium, there were no significant differences among the groups (*p* = 0.062, *η*^2^ = 0.131, 95% CI: [−0.036, 0.298]; Figure 3A), and the time factor was found to be significant (*p* < 0.001, *η*^2^ = 0.738, 95% CI: [0.688, 0.788]; Figure 3A). The serum sodium in N was significantly higher than that in W (139.67 ± 0.93 mmol/L vs. 138.35 ± 1.23 mmol/L; *p* = 0.010; Figure 3A). For serum potassium (*p* = 0.878, *η*^2^ = 0.013, 95% CI: [−0.177, 0.203]; Figure 3B) and chlorine (*p* = 0.109, *η*^2^ = 0.109, 95% CI: [−0.062, 0.280]; Figure 3C), there were no statistical differences between the four supplement strategies. The time factor for serum potassium (*p* = < 0.001, *η*^2^ = 0.551, 95% CI: [0.465, 0.636]; Figure 3B) and chloride (*p* < 0.001, *η*^2^ = 0.851, 95% CI: [0.822, 0.880]; Figure 3C) is significant, and this indicates that both indicators increased over time.

### 3.3. Muscle Damage and Recovery

**Figure 4:**
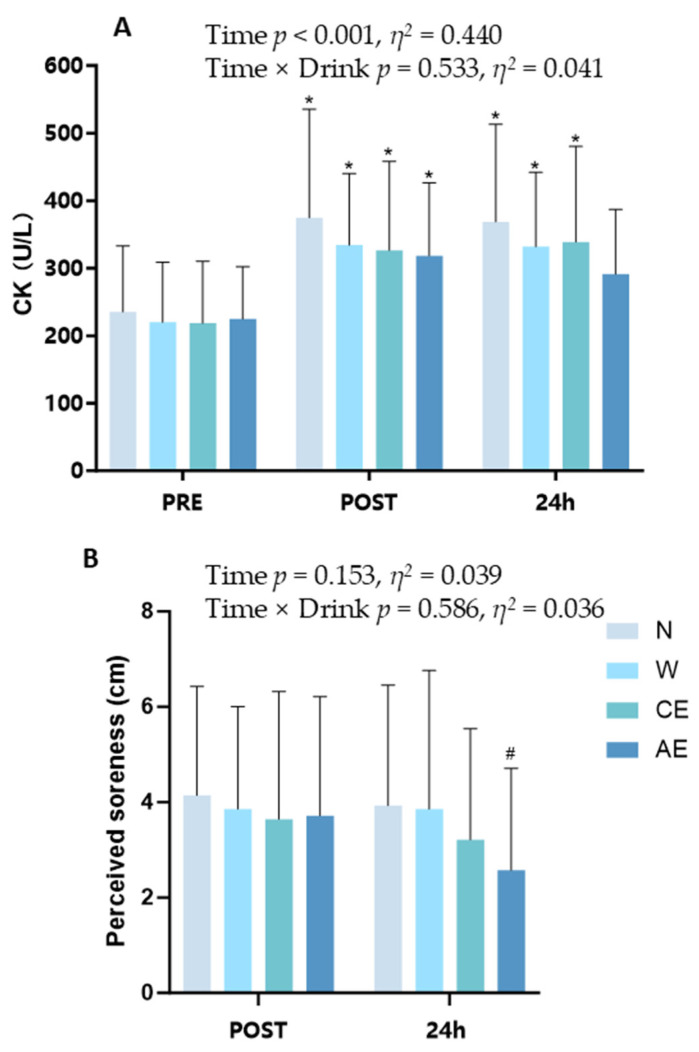
Analysis of muscle damage markers and muscle soreness after exercise: (**A**) serum creatine kinase concentration; (**B**) perceived muscle soreness ratings. Significance symbols: ^#^, significantly different from N, *p* < 0.05; *, significantly different from pre-exercise, *p* < 0.05.

Serum CK concentrations were significantly elevated above baseline in four supplement strategies post-exercise (*p* < 0.001, *η*^2^ = 0.440, 95% CI: [0.332, 0.548]; Figure 4A). Serum CK concentrations in N, W, and CE 24 h after exercise were significantly higher than pre-exercise CK levels (N: 235.46 ± 93.76 U/L vs. 368.81 ± 138.51 U/L; W: 220.13 ± 85.19 U/L vs. 331.97 ± 105.77 U/L; CE: 218.63 ± 88.06 U/L vs. 338.86 ± 136.00 U/L; *p* < 0.05; Figure 4A). However, there was no significant difference in CK levels between pre-exercise and 24 h post-exercise in AE (224.97 ± 74.20 U/L vs. 291.41 ± 91.79 U/L; *p* > 0.05; Figure 4A), and significantly less soreness in AE compared to N 24 h after exercise (2.6 ± 2.1 cm vs. 3.9 ± 2.4 cm; *p* < 0.05; Figure 4B).

### 3.4. Sweat Electrolyte and Electrolyte Balance

The sweat electrolyte concentration and whole-body sweat electrolyte loss displayed no difference between the four supplement strategies (Table 4). Sodium and chlorine losses greatly exceeded their intake, and there was no difference between the four supplement strategies in Na^+^ balance and Cl^−^ balance (Table 4). For K^+^ balance, AE was more positive compared to N, W, CE, and CE was more positive than W (*p* < 0.05, Table 4).

### 3.5. 5 km Performance and Muscle Strength

**Figure 5:**
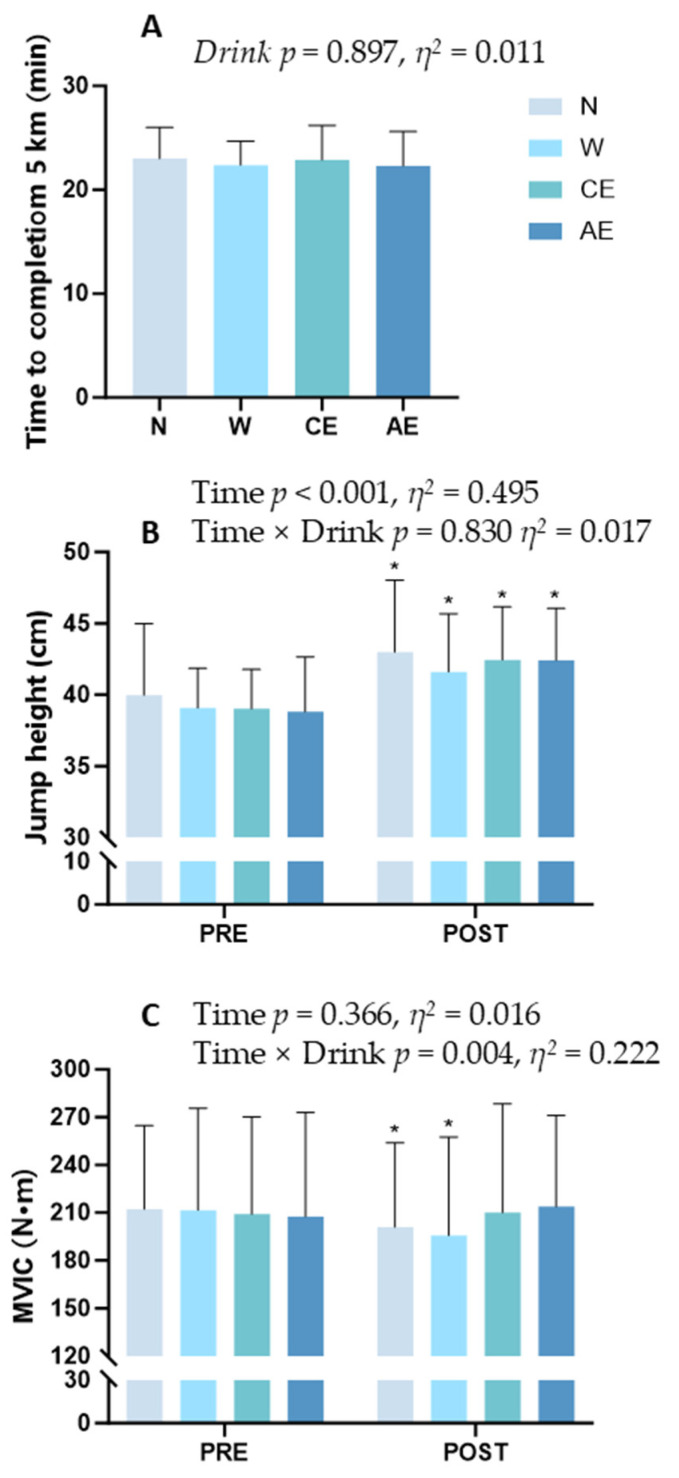
A 5 km time trial performance and muscle strength: (**A**) 5 km time trial performance; (**B**) maximal vertical jump height; (**C**) maximal voluntary isometric contraction output of quadriceps muscles (newton meters). Significance symbols: *, significantly different from pre-exercise, *p* < 0.05.

We observed no differences in 5 km performance between four supplement strategies (N: 23.00 ± 3.00 min; W: 22.40 ± 2.28 min; CE: 22.89 ± 3.32 min; AE: 22.31 ± 3.30 min; *p* = 0.897; *η*^2^ = 0.011, 95% CI: [−0.179, 0.201]; Figure 5A). Compared to pre-exercise, the vertical jump height significantly increased after exercise in all four supplement strategies (*p* < 0.001, *η*^2^ = 0.495, 95% CI: [0.398, 0.592]; Figure 5B), with no significant differences among the four supplement strategies (*p* = 0.830, *η*^2^ = 0.017, 95% CI: [−0.172, 0.206]; Figure 5B). For MVIC, we found differences between groups (*p* = 0.004, *η*^2^ = 0.222, 95% CI: [0.072, 0.372]; Figure 5C). In N and W, maximal voluntary isometric force output was significantly lower after exercise compared to pre-exercise (N: 212.21 ± 52.39 N·m vs. 200.93 ± 52.96 N·m; W: 211.57 ± 64.09 N·m vs. 195.79 ± 61.77 N·m; *p* < 0.05; Figure 5C). However, CE and AE showed no significant difference before and after exercise (CE: 209.14 ± 61.14 N·m vs. 210.14 ± 68.08 N·m; AE: 207.50 ± 65.32 N·m vs. 213.86 ± 57.23 N·m; *p* > 0.05; Figure 5C).

### 3.6. Perceptual Scales

**Figure 6:**
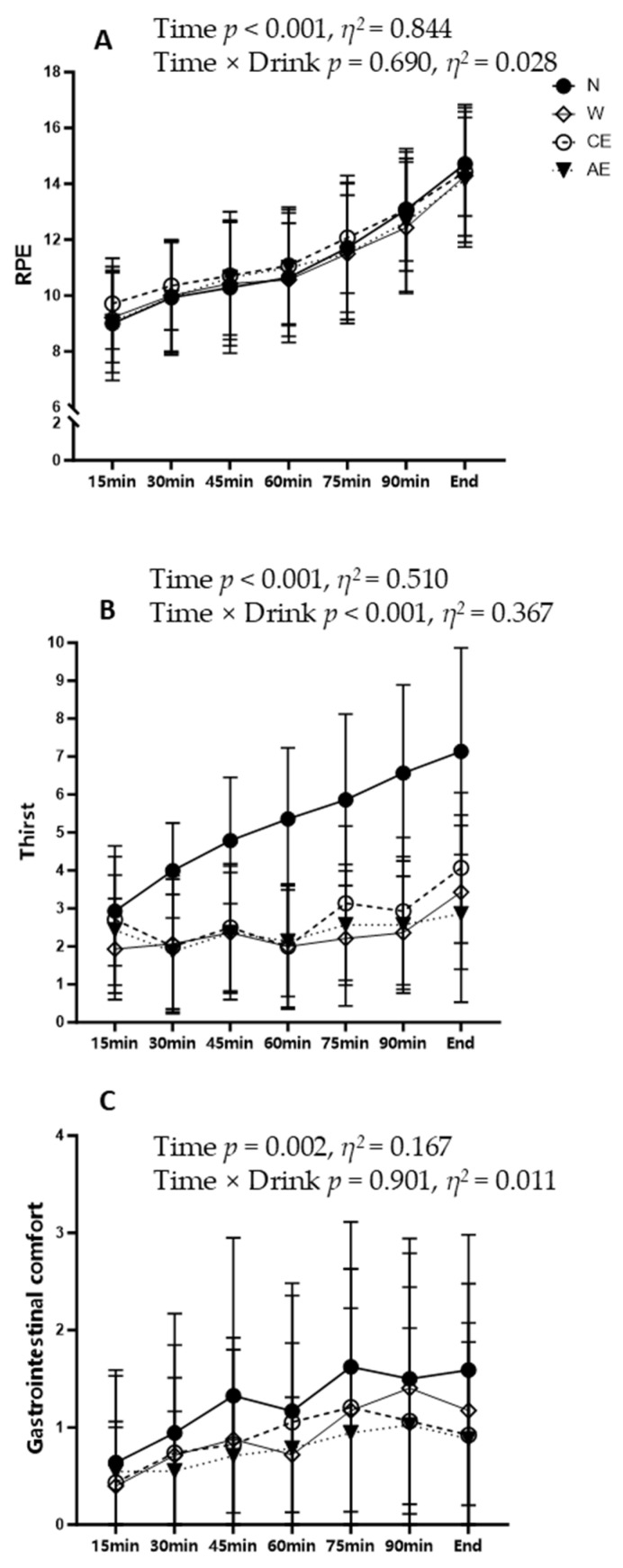
Perceptual responses during the experimental trials: (**A**) rating of perceived exertion; (**B**) rating of thirst; (**C**) rating of gastrointestinal comfort.

During the 21 km of running, RPE increased progressively with exercise duration, but no significant differences were observed among the four supplement strategies (Figure 6A). However, thirst levels in N increased over time, while W, CE, and AE were significantly lower than N (*p* < 0.05, Figure 6B). CE exhibited a notable increase in thirst from the beginning to the end of the exercise, while thirst levels in W and AE did not show a significant increase over time. For different fluid intakes, gastrointestinal comfort remained stable and did not show significant changes over time (Figure 6C).

## 4. Discussion

The purpose of this study was to examine the effects of a BCAAs-containing electrolyte beverage on hydration status, electrolyte balance, muscle damage, and performance, compared to a standard carbohydrate–electrolyte sports beverage and water. Participants completed four 21 km treadmill tests under controlled conditions (temperature: 23 ± 1 °C, relative humidity: 35 ± 5%). During each test, they supplemented with AE, CE, and W, or received no rehydration.

All participants experienced significant weight loss post-exercise, with the no-rehydration group losing about 3% of body weight and the fluid-supplemented groups losing around 2.5% (Figure 4A). The results for hydration status indicated that the percentage of nude body mass loss in the W, CE, and AE groups was significantly lower than that in the N group (*p* < 0.001; *η*^2^ = 0.382). Prolonged intense exercise can lead to fluid loss from both extracellular and intracellular compartments through sweating, resulting in a reduction in plasma volume [26]. Fluid supplementation during exercise helps maintain plasma volume. In this study, consuming AE experienced a smaller decrease in plasma volume compared to those in N (Cohen’s d = 0.76, Figure 2B). However, no significant differences in plasma volume changes were observed between W, CE, and N, which is possible because the amount of fluid consumed was far less than the fluid loss during exercise. Sodium transporters in the intestine allow for the co-transport of water and sodium promotes renal reabsorption of water, enhancing water absorption and reducing water loss, which helps maintain fluid balance and plasma volume [7,27]. Studies have shown that isotonic glucose–electrolyte beverages (high levels of electrolytes combined with low amounts of carbohydrates) can better enhance intestinal water absorption and support fluid balance during exercise compared to hypertonic beverages [4,28]. Additionally, electrolyte drinks with added amino acids can further promote water and sodium absorption [7,29,30,31]. The addition of small amounts of amino acids (3.48 g/L) to a sugar-free beverage increased fluid delivery to the circulation compared to a carbohydrate-based beverage [8]. AE demonstrated the best performance in maintaining plasma volume, which can be partially attributed to its isotonic nature, higher sodium content, and the addition of amino acids. In the experiment, we administered only a minimal volume of fluid, as it is inconvenient to hydrate frequently during a half marathon. This hydration approach mirrors the actual drinking habits of runners during races, making the experimental results more reflective of real-world conditions. In terms of running time, we found no significant differences in the latter-stage running performance among the four different fluid supplementation procedures. It is reported that exercise-induced dehydration (up to 4% body weight loss) does not alter aerobic performances during out-of-door exercise conditions [32]. Moreover, the loss of weight is conducive to reducing energy consumption and improving the running economy [33]. Athletes can perform well even with some level of dehydration, which could explain why there is often no noticeable difference in endurance performance whether they rehydrate or not. Supplementing with different types of fluids showed no effect on a 5 km time trial, which is consistent with findings from previous studies [6,34]. However, some studies have suggested that supplementing with BCAAs can enhance endurance performance [35,36]. Additionally, it is important to consider that factors such as pacing strategies, individual hydration habits, adaptations developed through training, and psychological influences may also play a role in performance during the final kilometers. Therefore, future studies should investigate these factors to provide a more comprehensive understanding of how hydration influences performance in endurance events.

The presence of BCAAs and protein in AE may provide some assistance in the recovery from muscle damage. After completing a half marathon, muscle damage biomarkers such as CK increased significantly [37]. Serum CK concentrations 24 h after exercise were significantly elevated in N, W, and CE, but not in AE (Figure 4A). Muscle soreness 24 h after exercise in AE was less than that in N (Figure 4B). This is consistent with findings from other studies indicating that BCAA supplementation can attenuate muscle damage and reduce muscle soreness [12,13,38,39]. The mammalian target of the rapamycin (mTOR) pathway is a cytoplasmic signaling pathway that controls cell growth and global metabolism, including protein and energy synthesis [40]. Evidence shows that BCAAs activate the mTOR pathway, promoting anabolic processes that accelerate muscle repair and reduce muscle damage caused by intense exercise. Controversially, some studies have reported that a dietary supplement of BCAAs alone cannot support an increased rate of muscle protein synthesis [41]. The high variability between studies—due to differences in training status, supplement dosage, timing of administration, and the severity of exercise-induced muscle damage (EIMD)—prevents us from conclusively determining whether BCAA supplementation via sports drinks during a half marathon is effective for runners. A systematic review and meta-analysis investigated that BCAA supplementation has a significant effect on the delayed onset of muscle soreness (DOMS) at 24 h and 48 h, but not immediately post-exercise [13]. Consistent with the results of this study, there were no significant differences in muscle damage markers immediately after exercise (Figure 4A). Co-ingestion of BCAAs and carbohydrates stimulates myofibrillar protein synthesis after resistance exercise [42]. Investigators have reported that consuming carbohydrate–protein beverages during exercise could attenuate post-exercise muscle damage compared to carbohydrate-only beverages [43,44]. However, the minimum effective dose of BCAAs required to reduce muscle damage remains undetermined [13]. Currently, there is no clear consensus on the effective minimum dose of BCAAs, with the existing literature providing a wide range of recommendations and significant individual variability. This gap underscores the need for further research to determine BCAA dosage requirements for different individuals and exercise types. While BCAA supplementation can enhance muscle recovery, increasing the dosage of a hydration beverage may reduce its overall effectiveness. Higher BCAA concentrations can increase viscosity, making the drink less palatable and potentially decreasing fluid intake. Thus, finding an optimal balance in BCAA dosing is essential to maintain both hydration and performance benefits. AE provides 1100 mg of BCAA in combination with 300 mcg of Vitamin B6 and other cofactors per serving. Vitamin B6 is essential for the transamination process of BCAAs, and a deficiency in this vitamin can hinder their breakdown [45]. Although the individual concentrations of BCAAs and Vitamin B6 in AE might not be high enough to elicit a significant ergogenic effect, this combination of ingredients could improve the utilization of BCAAs [46]. While the results of this study suggest the potential benefits of BCAAs-containing beverages, it is important to articulate their practical applications for athletes, coaches, and nutritionists. Incorporating BCAA beverages during training or competition may provide athletes with the necessary support to enhance recovery and performance. Additionally, adding an appropriate amount of BCAAs to regular hydration strategies could further optimize training outcomes. Future research should investigate the effectiveness of BCAAs-containing beverages across various scenarios, examining their role in athletes’ recovery during different training conditions and competitive settings. This exploration will help translate our findings into actionable recommendations for those in the field.

Electrolyte balance plays a critical role in maintaining physiological homeostasis during exercise, particularly for sustaining muscle function, nerve conduction, and fluid regulation. There were no significant differences among the four supplement strategies for Na^+^ balance and Cl^−^ balance, because the Na^+^ intake during exercise was much lower than the amount lost, resulting in a substantial negative Na^+^ balance (Table 4). In terms of K^+^ balance, AE demonstrated a significantly better K^+^ balance compared to N, W, and CE. This is because the net loss of K^+^ during exercise is smaller compared to sodium, and AE had a higher K^+^ content. The potassium balance in AE appeared to be optimal; however, there was no significant difference in serum potassium levels among the four supplement strategies. Since potassium is predominantly stored within intracellular fluid, it is possible that consuming AE beverages may better support intracellular potassium maintenance. Potassium efflux from the contracting muscle cell dramatically decreases the intracellular to extracellular potassium ratio, leading to depolarization of sarcolemma and t-tubular membranes [47]. All four groups showed a significant increase in serum Na^+^ concentration after 21 km of running, with W showing significantly lower serum Na^+^ levels compared to N (Figure 3A). The increase in serum Na^+^ concentration is due to substantial water loss through sweating during exercise, which decreases plasma volume and leads to higher sodium concentration in the blood. Dehydration can lead to hypernatremia, while excessive water supplementation can cause hyponatremia [48]. Therefore, electrolyte-containing beverages should be chosen for proper fluid replenishment during exercise. Furthermore, BCAAs can influence intracellular osmotic balance by regulating cell membrane permeability and water intake, thereby helping to maintain cell hydration. Additionally, they may affect osmotic pressure by increasing the intracellular protein content. This modulation of osmotic balance is crucial for preserving cellular function during various physiological conditions.

This study was conducted under controlled conditions. However, endurance events occur in variable outdoor environments. Therefore, future research should assess BCAAs-containing beverages in real-world settings. This will help determine their effectiveness under diverse environmental factors. While this study focused exclusively on trained young male athletes, the findings may limit the generalizability of the results to other populations, such as female athletes or untrained individuals. Acknowledging this limitation, future research should explore the long-term recovery effects of BCAA supplementation across diverse groups. Additionally, investigating varying dosage levels could provide insights into optimizing supplementation strategies. It is also essential to examine the responses of female athletes to BCAA intake, as hormonal and physiological differences may influence their effects on muscle recovery and performance. Such studies would enhance our understanding of BCAAs and inform tailored recommendations for different athlete populations.

## 5. Conclusions

Consuming a BCAAs-containing electrolyte beverage during a 21 km run can maintain electrolyte balance (particularly K^+^), reduce post-exercise muscle damage biomarkers (CK), and help to alleviate dehydration as well as the decline in lower limb strength following the run.

## Figures and Tables

**Table 1:** Participant characteristics.

Variable	Mean ± SD
Age (years)	22 ± 2
Height (cm)	176.6 ± 3.6
Body mass (kg)	67.2 ± 6.5
Body fat (%)	15.1 ± 3.8
VO_2_max (mL O_2_/kg/min)	55.2 ± 4.0

**Table 2:** AE and CE contents.

Amount per 100 mL	AE	CE
Calories (kJ)	85	102
Total carbohydrate (g)	4.7	6.0
Total protein (mg)	287	0
Peptide (mg)	67	0
BCAAs (mg)	220	0
Sodium (mg)	52	45
Potassium (mg)	39	5–25
Calcium (mg)	12	0
Magnesium (mg)	5	0
Niacin (mg)	0.6	0
Vitamin E (mg α-TE)	0.3	0
Vitamin B6 (mg)	0.06	0

**Table 3:** Total dietary intake during the experiment.

Dietary Intake	Mean ± SD
Total energy (kcal)	2176 ± 194
Protein (g)	109.4 ± 25.4
Protein caloric ratio (%)	20.01 ± 4.48
Carbohydrate (g)	254.8 ± 44.6
Fat (g)	81.8 ± 21.2
Sodium (g)	3.36 ± 0.60
Potassium (g)	1.94 ± 0.72

**Table 4:** Sweat electrolyte concentration and electrolyte balance.

	Variable	N	W	CE	AE
Sweat electrolyte concentration (mmol/L)	Na^+^	72.11 ± 21.02	71.29 ± 19.89	71.13 ± 18.06	69.69 ± 20.13
	K^+^	4.24 ± 0.52	4.31 ± 0.52	4.37 ± 0.52	4.29 ± 0.46
	Cl^−^	52.78 ± 18.62	52.52 ± 18.97	52.01 ± 16.09	51.47 ± 19.72
Na^+^ balance (mmol)	Na^+^ ingested	0	0	11.925	12.915
	Sweat Na^+^ loss	140.62 ± 39.93	143.02 ± 37.16	144.98 ± 40.05	140.53 ± 41.67
	Na^+^ balance	−140.62 ± 39.93	−143.02 ± 37.16	−133.05 ± 40.05	−127.62 ± 41.67
K^+^ balance (mmol)	K^+^ ingested	0	0	1.247	3.677
	Sweat K^+^ loss	6.55 ± 1.33	6.80 ± 1.25	6.94 ± 1.43	6.76 ± 1.34
	K^+^ balance	−6.55 ± 1.33	−6.80 ± 1.25	−5.69 ± 1.43 ^$^	−3.08 ± 1.34 ^#$&^
Cl^−^ balance (mmol)	Cl^−^ ingested	0	0	5.085	5.445
	Sweat Cl^−^ loss	113.35 ± 34.3	115.87 ± 34.42	116.95 ± 35.03	114.45 ± 39.18
	Cl^−^ balance	−113.35 ± 34.3	−115.87 ± 34.42	−111.86 ± 35.03	−109.01 ± 39.18

Legend: Sweat electrolyte concentration was obtained by analyzing sweat samples at the forearm. Electrolyte ingested was obtained by measuring the ion concentration in the beverage using an electrolyte analyzer. Sweat electrolyte loss (mmol) was calculated by multiplying sweat loss (L) by whole-body sweat electrolyte concentration (mmol/L). Sweat loss was calculated by adding body mass loss and fluid ingestion. Whole-body sweat electrolyte concentration was estimated by the prediction model for forearm sweat. Significance symbols: ^#^, significantly different from N, *p* < 0.05; ^$^, significantly different from W, *p* < 0.05; ^&^, significantly different from CE, *p* < 0.05.

## Data Availability

The original contributions presented in the study are included in the article, further inquiries can be directed to the corresponding author.
